# Extensive Diversity of RNA Viruses in Australian Ticks

**DOI:** 10.1128/JVI.01358-18

**Published:** 2019-01-17

**Authors:** Erin Harvey, Karrie Rose, John-Sebastian Eden, Nathan Lo, Thilanka Abeyasuriya, Mang Shi, Stephen L. Doggett, Edward C. Holmes

**Affiliations:** a Marie Bashir Institute for Infectious Diseases and Biosecurity, Charles Perkins Centre, School of Life and Environmental Sciences and Sydney Medical School, The University of Sydney, Sydney, Australia; b Australian Registry of Wildlife Health, Taronga Conservation Society Australia, Mosman, Australia; c Centre for Virus Research, Westmead Institute for Medical Research, Westmead, Australia; d School of Life and Environmental Sciences and Sydney Medical School, The University of Sydney, Sydney, Australia; e Department of Medical Entomology, NSWHP-ICPMR, Westmead Hospital, Westmead, Australia; Icahn School of Medicine at Mount Sinai

**Keywords:** *Ixodes holocyclus*, RNA virus, coltivirus, marsupial, phylogeny, ticks, virome

## Abstract

Understanding the microbiome of ticks in Australia is of considerable interest given the ongoing debate over whether Lyme disease and its causative agent, the bacterium Borrelia burgdorferi *sensu lato*, are present in Australia. The diversity of bacteria infecting Australian ticks has been studied using both culture- and metagenomics-based techniques. However, little is known about the virome of Australian ticks, including whether this includes viruses with the potential to infect mammals. We used a meta-transcriptomics approach to reveal the diversity and evolution of viruses from Australian ticks collected from two locations on the central east coast of Australia, including metropolitan Sydney. From this we identified 19 novel RNA viruses belonging to 12 families, as well as 1 previously described RNA virus. The majority of these viruses were related to arthropod-associated viruses, suggesting that they do not utilize mammalian hosts. However, two novel viruses discovered in ticks feeding on bandicoot marsupials clustered closely within the mammal-associated hepacivirus and pestivirus groups (family *Flaviviridae*). Another bandicoot tick yielded a novel coltivirus (family *Reoviridae*), a group of largely tick-associated viruses containing the known human pathogen Colorado tick fever virus and its relative, Eyach virus. Importantly, our transcriptomic data provided no evidence for the presence of B. burgdorferi *sensu lato* in any tick sample, providing further evidence against the presence of Lyme disease in Australia. In sum, this study reveals that Australian ticks harbor a diverse virome, including some viruses that merit additional screening in the context of emerging infectious disease.

**IMPORTANCE** Each year a growing number of individuals along the east coast of Australia experience debilitating disease following tick bites. As there is no evidence for the presence of the causative agent of Lyme disease, Borrelia burgdorferi *sensu lato*, in Australian ticks, the etiological basis of this disease syndrome remains controversial. To characterize the viruses associated with Australian ticks, particularly those that might be associated with mammalian infection, we performed unbiased RNA sequencing on 146 ticks collected across two locations along the coast of New South Wales, Australia. This revealed 19 novel RNA viruses from a diverse set of families. Notably, three of these viruses clustered with known mammalian viruses, including a novel coltivirus that was related to the human pathogen Colorado tick fever virus.

## Abstract (precis)

Each year a growing number of individuals along the east coast of Australia experience debilitating disease following tick bites. As there is no evidence for the presence of the causative agent of Lyme disease, Borrelia burgdorferi *sensu lato*, in Australian ticks, the etiological basis of this disease syndrome remains controversial. To characterize the viruses associated with Australian ticks, particularly those that might be associated with mammalian infection, we performed unbiased RNA sequencing on 146 ticks collected across two locations along the coast of New South Wales, Australia. This revealed 19 novel RNA viruses from a diverse set of families. Notably, three of these viruses clustered with known mammalian viruses, including a novel coltivirus that was related to the human pathogen Colorado tick fever virus.

## INTRODUCTION

Ticks are one of the most important vectors of infectious disease in humans, wildlife, and domestic animals (1), in part due to their ability to harbor multiple viruses, bacteria, and eukaryotic parasites, often simultaneously (2). Due to ecological changes affecting the life cycle and range of ticks, such as increased encroachment by humans on tick habitats and the impact of climate change in reducing tick mortality in winter and extending the active period, ticks are now an increasingly important vector of zoonotic disease globally (3). For example, cases of tick-borne bacterial infection, particularly Lyme disease, are becoming increasingly frequent across parts of North America and Europe (4, 5).

In Australia, Lyme disease is not recognized as endemic by the scientific or medical communities, although since the mid-1980s there has been considerable and high-profile controversy over its possible occurrence along the eastern coast of Australia (6–8). Importantly, the tick species known to act as a vector of Lyme disease are not found in Australia, and detailed studies involving microscopy, culturing, PCR, and metagenomic techniques indicate that the causative bacterial agent, Borrelia burgdorferi *sensu lato*, is not present in Australian ticks (6, 7). In addition, a North American strain of B. burgdorferi could not be transmitted by the Australian paralysis tick (Ixodes holocyclus) following experimental infection (9). Finally, and of most note, B. burgdorferi has not been conclusively detected in patients suffering clinical signs of disease following bites from Australian ticks (10). Hence, the cause of many human tick-borne disease events in Australia remains both unknown and controversial. Central to resolving this vexing public health issue is establishing an inventory of all the microbial species present in Australian ticks, particularly those that may be transmitted to humans.

A number of studies have used bacterial 16S rRNA-based metagenomics, as well as PCR- and immunoassay-based methods, to search for known or novel tick-associated bacterial pathogens in Australian ticks (11–13). Additional studies have used more powerful, bulk RNA sequencing (RNA-Seq)-based metagenomics approaches to identify viruses in diverse tick populations, although not those from Australia (14). Although it is now clear that ticks carry a diverse virome and are associated with the transmission of viral pathogens in parts of Europe, North America, Asia, and Africa (15–19), there has been no comprehensive metagenomic study of the virome of ticks in Australia.

Australia and its wildlife have largely evolved in isolation for over 46 million years and are characteristically unique and diverse. Hence, a survey of tick viruses in the context of tick-borne disease might be expected to identify equally diverse and unexpected organisms (20). Indeed, a number of novel tick-borne viruses, such as Albatross Island virus and Saumarez Reef virus, have been identified using culture-dependent methods in Australian wildlife (21, 22). However, these methods are inherently limited by the number of viruses that can be cultured and have often focused on microbial isolation following die-off events in the ticks’ host populations. The description of such a small number of tick-associated viruses means that there is a paucity of knowledge about the natural virome of ticks in Australia.

We have recently shown that meta-transcriptomics (i.e., bulk shotgun RNA sequencing) is a powerful tool for the discovery of novel viruses and other microbial pathogens from both host- and tick-derived samples (14, 23–25). Importantly, because total RNA is sequenced, meta-transcriptomics provides information on the total infectome of the sample and can be used to identify viruses, bacteria, fungi, and other eukaryotes present within a sample (26). The method also provides quantitative data on virus abundance that may be used to identify potential pathogens (26). To better understand the natural virome of Australian ticks and their evolutionary relationships, with a specific focus on RNA viruses, we performed unbiased RNA sequencing on 146 ticks from three species of tick collected from native lizards, marsupials, and rodents and from introduced rodents, as well as from unfed nymphal ticks. In particular, we sought to determine whether Australian ticks harbor any viruses that are from families and/or genera previously associated with human and other mammalian disease and, hence, that could feasibly contribute to tick-borne disease in Australia. Tick specimens were collected from the North Shore region of Sydney and the South Coast region of New South Wales (NSW), and the data obtained provide important insights into the remarkable diversity of tick-associated RNA viruses.

(This article was submitted to an online preprint archive [27].)

## RESULTS

### Assignment of tick species.

**FIG 1:**
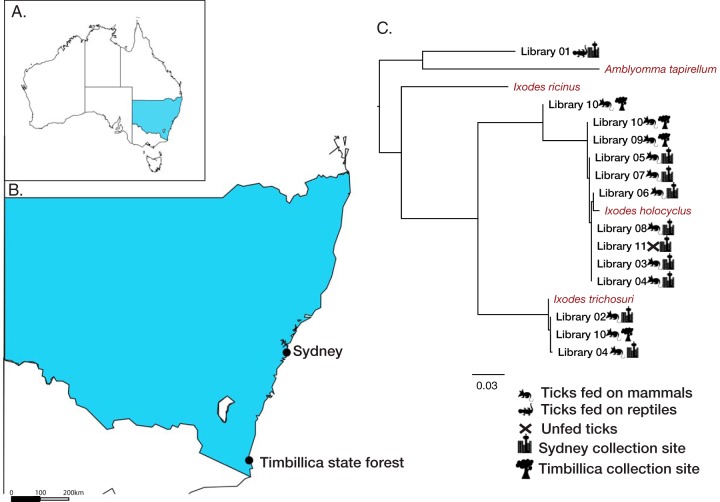
(A) Map of Australia showing the location of New South Wales, Australia, where sampling took place. (B) Locations of tick sampling sites, marked by solid black dots on a map of New South Wales. (C) Maximum likelihood phylogeny of the mitochondrial cytochrome *c* oxidase subunit I (COX1) gene from each pooled library, with the sampling species (and unfed ticks) and location indicated. Reference COX1 sequences from a variety of tick species are shown in red. The scale bar indicates the number of nucleotide substitutions per site. The tree was midpoint rooted for clarity only.

In total, 146 ticks were collected across two locations in New South Wales, Australia, during 2016 and 2017. The samples represented three tick species common to the east coast of Australia, Ixodes holocyclus, Ixodes trichosuri, and Amblyomma moreliae (Table 1). Ticks were pooled into 11 libraries based on the tick species, collection location, host species, and life cycle stage, where possible (Table 1). RNA sequencing generated between 32,280,822 and 59,377,716 reads per library, which were assembled *de novo* into between 159,626 and 505,268 contigs per library (Table 1). Tick mitochondrial COX1 sequences were identified from the assembled contigs and used to confirm the species identification of the ticks in each library (Fig. 1).

**TABLE 1:** Summary details of each RNA sequencing library

Library	Tick species	Tick life cycle stage	Tick host	Collection location	No. of individuals	No. of reads
1	Amblyomma moreliae	Adult	Blue-tongue lizard	Sydney	20	54,810,238
2	Ixodes trichosuri	Adult	LNB,^*a*^ Bush rat	Sydney	3	56,647,294
3	Ixodes holocyclus	Adult	Human	Sydney	1	59,377,716
4	Ixodes holocyclus	Nymph	LNB, Black rat	Sydney	11	54,145,842
5	Ixodes holocyclus	Adult	LNB	Sydney	6	59,256,412
6	Ixodes holocyclus	Adult	LNB	Sydney	9	57,976,012
7	Ixodes holocyclus	Adult	LNB	Sydney	5	55,605,784
8	Ixodes holocyclus	Adult, nymph	LNB	Sydney	9	32,280,822
9	Ixodes holocyclus	Adult, nymph	SBB^*b*^	Timbillica	9	34,448,236
10	Ixodes holocyclus	Adult, nymph	SBB	Timbillica	15	40,034,328
11	Ixodes holocyclus	Nymph	Unfed	Sydney	58	37,670,822

a LNB, long-nosed bandicoot.

b SBB, southern brown bandicoot.

A single COX1 sequence was identified for libraries 3, 5 to 9, and 11 and was closely related to the I. holocyclus COX1 reference sequence taken from NCBI (Fig. 1). In contrast, libraries 4 and 10 contained multiple distinct COX1 sequences, suggesting that there may have been ticks of more than one species included in the library that had been incorrectly classified. No reference sequence was available for A. moreliae ticks. Accordingly, the most closely related tick species available in the NCBI RefSeq database, Amblyomma tapirellum, was included in the phylogeny as a proxy, although it is important to note that A. tapirellum is not found in Australia. Notably, none of the COX1 sequences identified in the 11 transcriptomes showed strong sequence similarity to the sequence of Ixodes ricinus, the main vector of B. burgdorferi *sensu lato* in western Europe, or to the sequences of Ixodes scapularus, Ixodes persulcatus, or Ixodes pacificus ticks.

### The tick virome.

Overall, the complete coding sequences of 16 viral genomes were identified within the tick data set collected here using a series of BLAST searches based on protein sequence similarity. An additional four partial viral genomes were also identified. Of these 20 complete or partial genomes, 19 contained previously undescribed viruses, although all fell within known viral families or orders, specifically: *Mononegavirales*, *Chuviridae*, *Orthomyxoviridae*, *Picornaviridae*, *Flaviviridae*, *Narnaviridae*, *Luteoviridae*, *Virgaviridae*, *Reoviridae*, *Phenuiviridae*, and *Partitiviridae*. All viruses identified across the 11 libraries were RNA viruses with no DNA intermediate, but the full diversity of RNA virus genome structures was represented, that is, positive-sense single-stranded RNA (+ssRNA), negative-sense single-stranded RNA (−ssRNA), and double-stranded RNA (dsRNA), segmented, and nonsegmented. It is unlikely that any of these viruses are endogenous viral elements present within the tick genome, as they were identified by open reading frames (ORFs) unbroken by stop codons, and none of the nucleotide sequences showed similarity to tick gene sequences within the NCBI BLAST database. All novel viruses identified in this study were named after locations and landmarks within the tick collection areas.

**FIG 2:**
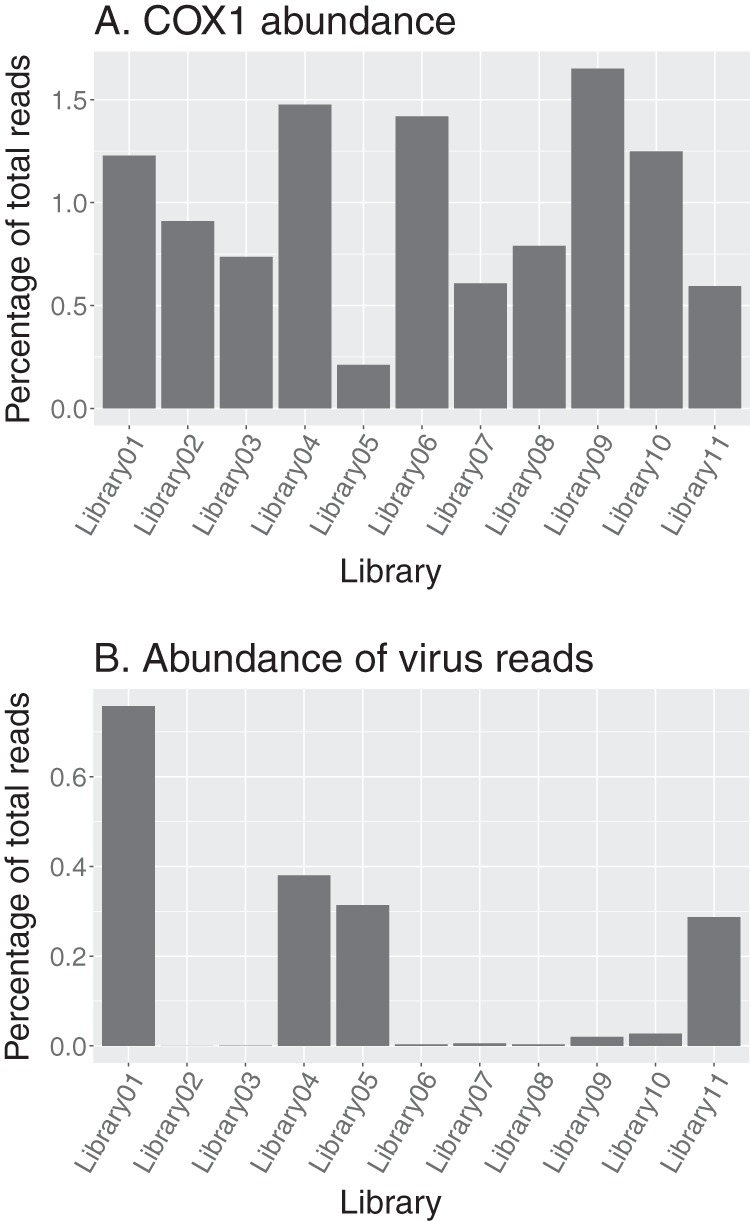
(A) Percentage of reads aligning to the tick mitochondrial COX1 reference sequence. (B) Percentage of reads aligning to RNA virus genomes in each library. The library composition is described in Table 1.

The housekeeping gene COX1 was used to measure the abundance of the invertebrate (i.e., likely host) transcripts in each library. Accordingly, the COX1 abundance ranged from 0.15% to 1.1% of the total number of reads (from which rRNA was already excluded; Fig. 2). We also calculated the proportion of virus reads as a fraction of total reads. This revealed that while the number of viral reads in each library ranged from 47 to 268,775 (0.00017% to 0.29% of the number of nonribosomal reads within a library), it was not related to the abundance of host genes (Fig. 2). However, the number of virus species represented in each library, which varied from one to six, appeared to be associated with the percentage abundance of virus reads (Fig. 2).

**FIG 3:**
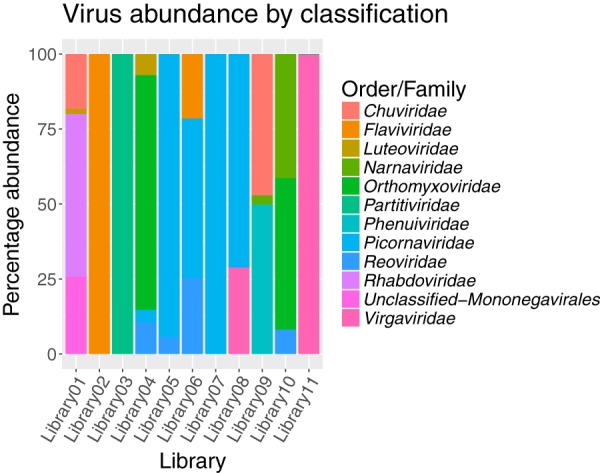
Abundance of viruses from each family or order, shown as the percentage of the total number of viral reads in each library.

Viruses from the newly classified *Chuviridae* family (−ssRNA) and the *Picornaviridae* (+ssRNA) were the most abundant, while viruses from the *Flaviviridae* (+ssRNA) were the least abundant. In libraries containing multiple viruses, the abundance of each species varied substantially. For example, in library 1, Manly virus represented 54% of the total number of virus reads, while Quarantine Head virus represented only 0.9% of the total virus reads in that library (Fig. 3).

The evolutionary history of each novel virus was investigated using phylogenetic analysis. In particular, this analysis was used to infer both the relationship of each virus to previously characterized viruses and its likely host. The 19 novel viruses described here showed various degrees of similarity to those previously identified.

### Positive-sense RNA viruses.

**FIG 4:**
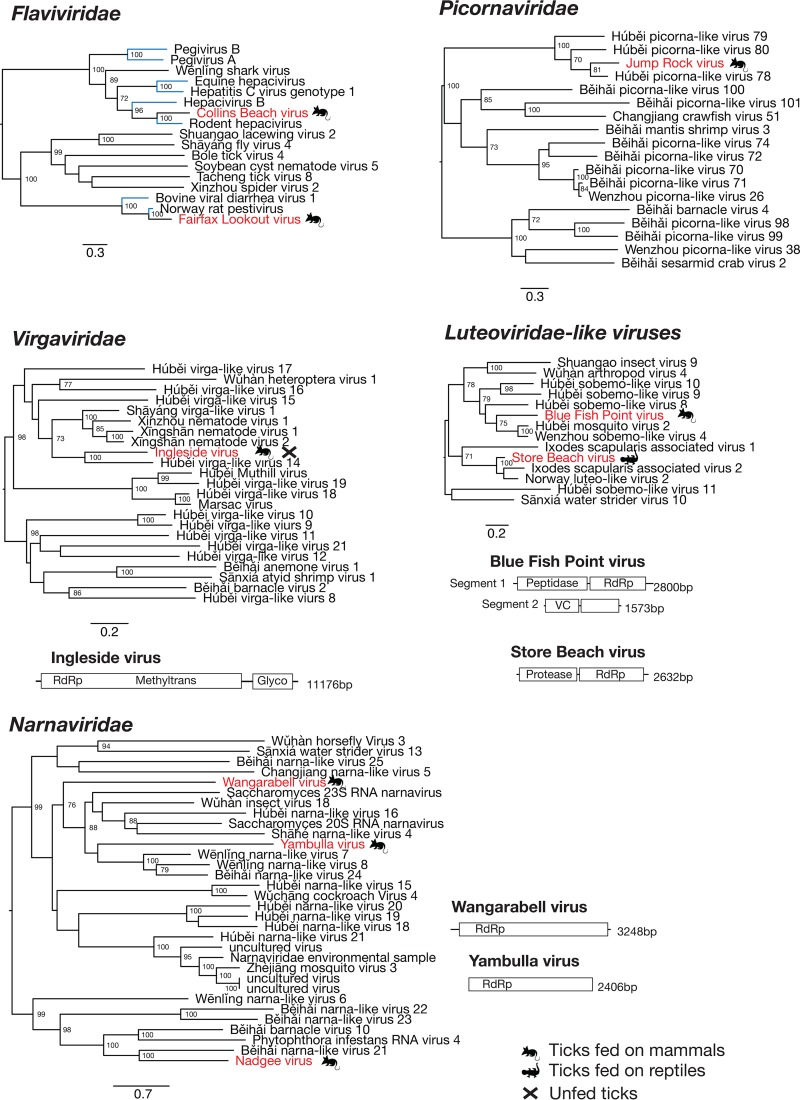
Phylogenetic relationships and genomic structure of the +ssRNA viruses sampled in this study. All trees are scaled according to the number of amino acid substitutions per site. The trees are midpoint rooted for clarity only, and bootstrap values (>70%) are shown. Viruses determined here are shown in red. The symbols denote the vertebrate host that the tick was taken from or whether the virus was sampled from an unfed tick. Virus lineages previously shown to infect mammals or mammalian cells are shown in blue. Genome diagrams provide information on the length of each genomic segment, the number of ORFs, and predicted conserved protein structures. No diagrams are provided for viruses for which no full genomes were identified.

Our data contained genomic evidence for the presence of nine +ssRNA viruses belonging to five virus families, representing just under half of the viruses identified in this study. Importantly, this includes two flavi-like viruses potentially associated with mammalian hosts, rather than the tick itself, based on their position within the *Flaviviridae* phylogeny (Fig. 4) and their low abundance (Fig. 2). Specifically, both Collins Beach virus and Fairfax Lookout virus exhibited an extremely low abundance in the data set, and only partial viral genomes were identified (Table 2). Despite this, enough of the viral genome was assembled to perform robust phylogenetic analysis, which revealed that these viruses are closely related to rodent hepacivirus and Norway rat pestivirus, respectively (Fig. 4). Their relatively close evolutionary relationship to other rodent-associated viruses gives additional support to the idea that these viruses are associated with the blood meal contained within the engorged ticks and may not be infecting (i.e., replicating in) the ticks themselves. Notably, Fairfax Lookout virus was identified in I. trichosuri ticks that had fed on long-nosed bandicoots (Perameles nasuta; a marsupial) and native bush rats (Rattus fuscipes) (Table 1). Similarly, Collins Beach virus was found in I. holocyclus ticks collected from long-nosed bandicoots (Table 1).

**TABLE 2:** Name and description of novel viruses in Australian ticks

Virus name	Abbreviation	Virus family/order	Genome length/contig length (nt)	No. of segments	Library(ies)	Complete genome^*a*^
Manly virus	MLYV	*Rhabdoviridae*	9,835	1	1	Y
Fairlight virus	FLTV	*Mononegavirales*	9,047	1	1	Y
Cannae Point virus	CPTV	*Chuviridae*	11,192	1	1	Y
Store Beach virus	STRBV	Luteo-like virus	2,632	1	1	Y
Quarantine Head virus	QHV	*Mononegavirales*	8,667	1	1	Y
North Shore virus	NTSV	*Partitiviridae*	1,770	1	3, 5, 11	Y
Blue Fish Point virus	BFPV	Luteo-like virus	4,373	2	4	Y
Shelly Headland virus	SHLV	*Reoviridae*	26,411	11	5, 6	Y
Jump Rock virus	JRV	*Picornaviridae*	5,202	1	5	N
Ingleside virus	INGV	*Virgaviridae*	11,176	1	8, 11	Y
Collins Beach virus	CBV	*Flaviviridae*	1,343	1	5, 6	N
Fairfax Lookout virus	FFLV	*Flaviviridae*	2,194	1	2	N
Timbillica virus	TBLV	*Phenuiviridae*	8,909	2	9	Y
Genoa virus	GEV	*Chuviridae*	11,018	1	9	Y
Nadgee virus	NDGV	*Narnaviridae*	1,733	1	9	N
Wangarabell virus	WBV	*Narnaviridae*	3,248	1	10	Y
Yambulla virus	YMBV	*Narnaviridae*	2,406	1	10	Y
Old Quarry Swamp virus	OQSV	*Orthomyxoviridae*	10,498	5	4, 10	Y
Shelly Beach virus	SHLBV	*Reoviridae*	13,739	6	4, 10	Y

a Y, yes; N, no.

In contrast, all the remaining +ssRNA viruses identified were closely related to arthropod-associated viruses. Ingleside virus fell within the virga-like virus cluster and was most closely related to a virus found in spiders from China (Fig. 4) (23). The genome of Ingleside virus is 11,176 nucleotides (nt) long and encodes two predicted ORFs. This virus represented 99.8% of viral reads and 0.2% of the total reads in library 11 and was also present at a low abundance in library 8 (Table 2; Fig. 2). Similarly, Blue Fish Point virus and Store Beach virus clustered within the *Luteoviridae*. While Blue Fish Point virus, a bisegmented virus with 4 ORFs, was most closely related to mosquito-associated viruses, Store Beach virus appeared to comprise a single segment with two ORFs that grouped closely with other *Ixodes*-associated viruses (Fig. 4). These viruses were also sampled from different ticks. Blue Fish Point virus was associated with I. holocyclus ticks collected from long-nosed bandicoots, while Store Beach virus was identified in lizard ticks and was the only virus found in the Amblyomma moreliae pool. Despite the divergent phylogenetic position of A. moreliae, Store Beach virus was closely related to viruses associated with *Ixodes* ticks (Fig. 4). Finally, Jump Rock virus grouped with other picorna-like viruses identified in Chinese arthropods (Fig. 4) (23). A full genome of this virus was not recovered from the data, likely due its low abundance, as the 5,202-bp fragments represented only 0.14% of viral reads in the library.

Viruses showing similarity to the narna-like virus group were identified only in ticks collected in southern NSW (Timbillica), and all three viruses were extremely divergent from previously described viruses. The amino acid sequence similarity to the most closely related virus species ranged from 24 to 32% for these three viruses across the RNA-dependent RNA polymerase (RdRp) region. The genomes of these viruses comprised a single genome segment with one predicted ORF containing an RdRp-like region, typical of viruses within this group, which have no known structural proteins (28). Complete coding regions were recovered for two of these viruses, designated Wangarabell virus and Yambulla virus, while only fragments of Nadgee virus were recovered from this data set.

The previously identified Ixodes holocyclus iflavirus (29), found in I. holocyclus ticks using a viral RNA isolation method, was present in five libraries (libraries 4 to 8). This was the only previously described virus found in any of the 11 data sets generated here. This virus was identified only in I. holocyclus ticks collected in the Sydney region.

### Double-stranded RNA viruses.

**FIG 5:**
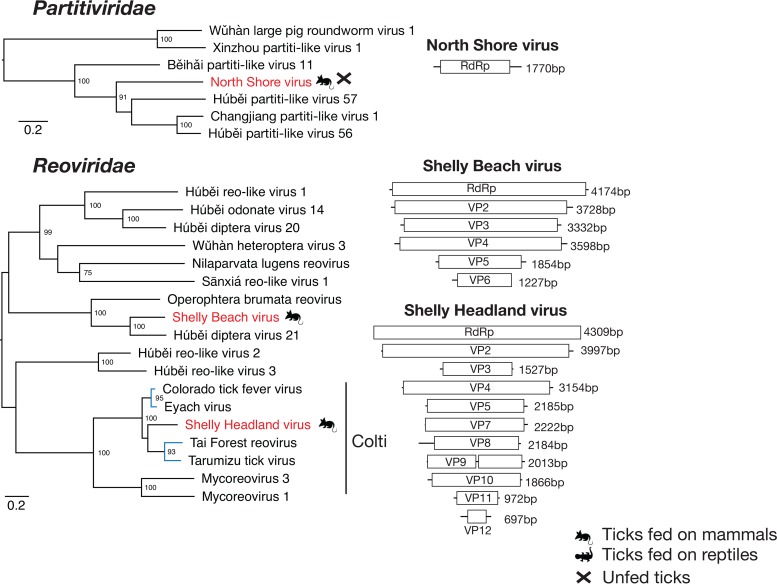
Phylogenetic relationships and genomic structure of the dsRNA viruses sampled in this study. All trees are scaled according to the number of amino acid substitutions per site. The trees were midpoint rooted for clarity only, and bootstrap values (>70%) are shown. Viruses determined here are shown in red. The symbols denote the vertebrate host that the tick was taken from or whether the virus was sampled from an unfed tick. Virus lineages previously shown to infect mammals or mammalian cells are shown in blue. Genome diagrams provide information on the length of each genomic segment, the number of predicted ORFs, and predicted conserved protein structures.

Our data contained genomic evidence for the presence of three dsRNA viruses from two families. North Shore virus clustered with uncharacterized partiti-like viruses isolated from arthropods. Our phylogenetic analysis revealed that this group of viruses fell between the deltapartitiviruses and the gammapartitiviruses, which are known to infect plants and fungi, respectively (Fig. 5). For this reason, and because partitiviruses are known to infect a wide range of hosts from protozoans to fungi (30), it was impossible to determine the likely host of these viruses. A single segment with a single ORF showed only approximately 50% amino acid sequence similarity over the conserved RdRp region. This virus was found in three libraries of I. holocyclus ticks from the Sydney region (Table 1).

In contrast, Shelly Beach virus and Shelly Headland virus clustered with the *Reoviridae*, a family of segmented RNA viruses (Fig. 5). Shelly Beach virus was found to have six segments encoding six ORFs and was most closely related to other reoviruses associated with flies and moths (23). This was one of two viruses found in I. holocyclus ticks collected from both locations. Of greater interest was Shelly Headland virus, which grouped with the subfamily of tick-associated coltiviruses. Importantly, two coltiviruses have been implicated in human disease in North America and Europe: Colorado tick fever virus (CTFV) and Eyach virus (15, 16). The other two members of the genus were recently identified in viral culture of samples isolated from ticks (Haemaphysalis flava) in Japan and the blood of free-tailed bats (Chaereophon aloysiisabaudiae) in Côte d’lvoire (31, 32). In total, 11 segments of Shelly Headland virus containing 12 ORFs were identified, with segment 9 containing two predicted ORFs separated by a single stop codon. It is currently unclear if there is a 12th segment containing a viral protein similar to that of viral protein 6 in CTFV that cannot be identified through amino acid sequence similarity or if this segment is not present in this virus.

### Negative-sense RNA viruses.

**FIG 6:**
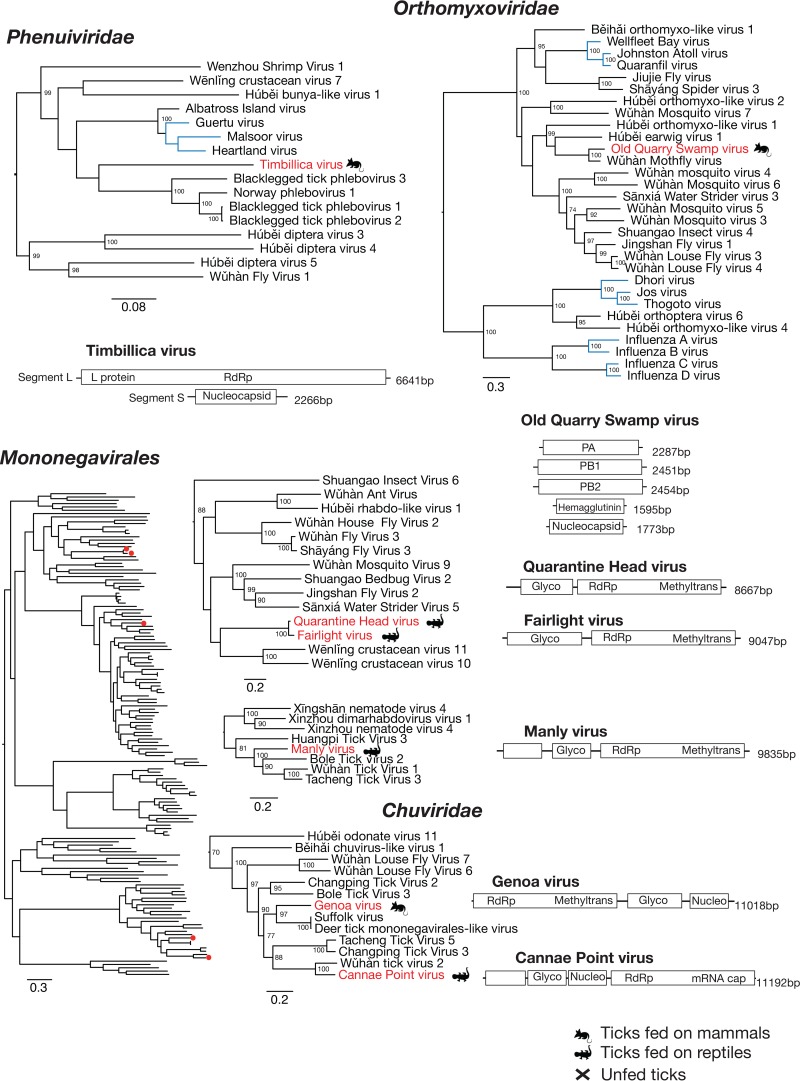
Phylogenetic relationships and genomic structure of the −ssRNA viruses sampled in this study. All trees are scaled according to the number of amino acid substitutions per site. The trees are midpoint rooted for clarity, and bootstrap values (>70%) are shown. Viruses determined here are shown in red. The symbols denote the vertebrate host that the tick was taken from or whether the virus was sampled from an unfed tick. Virus lineages previously shown to infect mammals or mammalian cells are shown in blue. Genome diagrams provide information on the length of each genomic segment, the number of predicted ORFs, and predicted conserved protein structures.

The complete coding region of seven −ssRNA viruses were identified within 4 of the 11 libraries produced in this study. Two of these viruses fell within the *Phenuiviridae* and *Orthomyxoviridae*, while the remaining five fell within the order *Mononegavirales* and clustered within the *Chuviridae* (33). Although Timbillica virus fell within the *Phenuiviridae*, it was extremely divergent from previously described viruses in this family, exhibiting only 36% amino acid sequence similarity to the most closely related virus (blacklegged tick phlebovirus 3). The most closely related viruses were also tick associated and were isolated from ticks in Norway and North America (Fig. 6) (14, 34). Also of note was the finding that Timbillica virus grouped more closely with viruses associated with *Ixodes* ticks rather than with Albatross Island virus (previously known as Hunter Island virus), identified in avian ticks collected from albatross in Tasmania (21). However, only two segments with a single ORF each were identified (Fig. 6). This could be due to either an absence or a lack of identification through amino acid sequence similarity searches of the third segment typically associated with phleboviruses. Old Quarry Swamp virus was the only virus identified in both the Timbillica and Sydney ticks, although the virus was far more abundant in the library containing ticks from the Sydney region (Fig. 3). Interestingly, this virus was most closely related to other arthropod-associated viruses and contained at least five segments, each with a single ORF, as was typical of other related viruses (Fig. 6).

The most well-represented order of viruses discovered in our survey was the *Mononegavirales*. Specifically, we identified two novel viruses clustering within the recently described *Chuviridae* family of arthropod-associated viruses (23), one within the *Rhabdoviridae*, and two that clustered with a number of unclassified arthropod-borne viruses. These viruses were often at a relatively high abundance in the libraries in which they were identified.

Genoa virus and Cannae point virus clustered with the *Chuviridae*, within a group containing Suffolk virus, deer tick *Mononegavirales*-like virus, and Wuhan tick virus 2 (Fig. 6). Genoa virus and Cannae Point virus showed relatively close evolutionary relationships to previously described tick-associated viruses, with the former clustering with viruses associated with Ixodes scapularis ticks and the latter clustering with viruses identified in Rhipicephalus microplus ticks (33, 34). Genoa virus represented ∼47% of total virus reads in the library in which it was identified, while Cannae Point virus represented ∼18% of the viral reads identified in library 1. Both viruses contained a single segment, although the Genoa virus genome encoded three ORFs, while the Cannae Point virus genome encoded four (Fig. 6). Manly virus was closely related to other tick-associated viruses within the *Rhabdoviridae* and represented 54% of the total viral reads in library 1 and 0.26% of the total reads in the library. The genome is a single segment approximately 9,835 nt in length that encodes three ORFs. Quarantine Head virus and Fairlight virus exhibited a close evolutionary relationship, with 95.7% amino acid sequence identity between the two RdRp sequences. These viruses were highly divergent from those previously identified, and their sampled relatives are known to infect crustaceans and are as yet unclassified (23). Fairlight virus represented approximately 25% of total virus reads, while Quarantine Head virus represented only approximately 1% of viral reads in this library. Both viral genomes encoded two ORFs.

### Identification of nonviral microbial organisms.

The largest diversity of microbial species other than viruses (i.e., bacteria, fungi, and single-cell eukaryotes) was present in library 1 (Table 3). However, it is important to note that this analysis considered only those sequences that could be confidently identified using a Diamond BLAST search against the NCBI nr protein sequence database. All other libraries contained “*Candidatus* Midichloria mitochondrii” exclusively, aside from library 5, which also contained an *Enterobacter* sp., trypanosomes, and Kluyvera intermedia (Table 3). Finally, it is important to note that no reads matching the bacterium B. burgdorferi *sensu lato* were identified in any of the BLAST searches of the RNA-Seq data generated here.

**TABLE 3:** Bacterial, fungal, and single-cell eukaryotes identified in the RNA-Seq data

Host organism	Identification of contigs in the following library:										
	1	2	3	4	5	6	7	8	9	10	11
Absidia glauca	**+**										
Francisella persica	**+**										
Phycomyces blakesleeanus	**+**										
Rhizopus oryzae	**+**										
Hesseltinella vesiculosa	**+**										
Stegodyphus mimosarum	**+**	**+**	**+**	**+**	**+**	**+**	**+**	**+**	**+**	**+**	**+**
Fusarium graminearum	**+**										
Fusarium oxysporum	**+**										
“*Candidatus* Midichloria mitochondrii”		**+**	**+**	**+**	**+**	**+**	**+**	**+**	**+**	**+**	**+**
*Enterobacter* sp.					**+**						
*Trypanosoma* sp.					**+**						
Kluyvera intermedia					**+**						

## DISCUSSION

We present the first metagenomic description of the viruses present in Australian ticks. Analysis of the transcriptomes of 146 ticks collected from two locations on the NSW coast revealed the capacity of these ticks to harbor a wide diversity of novel viruses. Previous meta-transcriptomic studies targeting arthropod viromes have revealed an abundance of novel and often highly divergent viruses (14, 23–25, 33). To date, however, there has been no study of the diversity of tick-associated viruses in Australia or of whether any of these viruses might play a role in tick-borne disease, despite the controversy surrounding tick-borne disease in this country (6, 7). Importantly, we also used the RNA-Seq meta-transcriptomic data generated here to search for the presence of known tick-associated pathogens, particularly bacteria of B. burgdorferi *sensu lato*, the complex of bacterial species causing Lyme disease in parts of Europe and North America. Although a number of bacterial, fungal, and eukaryotic species were identified within our data, no B. burgdorferi *sensu lato* bacteria were detected. This is consistent with the findings of recent bacterial 16S rRNA-based metagenomic studies performed with larger samples of Australian ticks (11, 12). Hence, our results are in accord with the growing consensus that Lyme disease is not present in Australia.

The abundance of the mitochondrial COX1 gene was not uniform across all libraries, although this is expected due to such factors as variation in the number of ticks included in each library, the size of each individual tick, and the extent to which the tick was engorged. It may also reflect the differing abundance of bacterial and fungal genetic material in the library. Importantly, however, the libraries were constructed based on the tick’s collection location, species, and host and the amount of RNA extracted from that tick, so that approximately equal amounts of RNA from each tick were included within a library.

Overall, we identified 19 novel viruses, one of which clearly merits additional investigation as a potential zoonotic pathogen based on its phylogenetic relationship to other tick-borne viruses associated with human disease. Specifically, Shelly Headland virus fell within the genus *Coltivirus* (*Reoviridae*), which currently comprises four virus species, two of which have been implicated in human tick-associated disease: Colorado tick fever virus and Eyach virus in North America and Europe, respectively (15, 16). Coltiviruses have also been isolated from rodents and bats, as well as in ticks that act as transmission vectors (31, 32). It is therefore possible that Shelly Headland virus infects the long-nosed bandicoots which represent a distinct mammalian host, and may have the potential to cause zoonotic disease. This would obviously be of considerable concern given the close proximity of dense urban development to the national park in which these long-nosed bandicoots are found in Sydney. A systematic screening for Shelly Headland virus in long-nosed bandicoots and other mammalian species, as well as in human cases of tick-borne disease in Australia, is therefore warranted.

We also identified two novel viruses belonging to the *Flaviviridae* that clustered closely with rodent-associated viruses. This phylogenetic position, in combination with the extremely low abundance of these viruses, suggests that they were in fact derived from the tick’s vertebrate host and were present in the blood contained in the engorged tick rather than being from the tick itself, although this will need additional verification.

The majority of the ticks sampled and sequenced in this study were collected from national parks in the Sydney region, the most populated area in Australia. Encroachment of urban development on the habitats of native wildlife not only threatens the survival of native flora and fauna but, by changing the nature of the human-animal interface, may also increase the risk of novel zoonotic disease. For example, changes in land use and the encroachment of human development on bat habitats in subtropical areas of Australia have resulted in increased contact between fruit bats and horses, in turn leading to a spillover of Hendra virus into the equine population (35). On a number of occasions, Hendra virus has been transmitted from horses to humans and resulted in fatal disease outcomes (36).

Of note was that Timbillica virus groups with several other tick-associated viruses within the *Phenuiviridae*, falling within the newly classified genus *Banyangvirus*, and is related (albeit distantly) to Heartland virus that has been implicated in human disease in North America (37). Interestingly, another virus within this group, Albatross Island virus, was identified as a potential cause of a disease outbreak in the shy albatross (Thalassarche cauta) in northwestern Tasmania, Australia (21). The only previously described virus found within our data was the picornavirus Ixodes holocyclus iflavirus. This virus was previously identified in the salivary glands of I. holocyclus ticks collected in the coastal regions of northern NSW and southern Queensland (29), and we detected it in the ticks collected from the Sydney region.

In sum, using a powerful meta-transcriptomics approach, we identified 19 novel and sometimes divergent viruses within ticks collected in the central east coast of Australia, one of which is related to those already known to cause human disease. It is therefore clear that there is a great diversity of viruses in Australian wildlife that are yet to be discovered, and as contact between urban areas and native wildlife increases, it is possible that some of these may pose a risk to public health and, hence, require careful monitoring.

## MATERIALS AND METHODS

### Ethics statement.

Ethics approval for this work was granted by the NSW Office of Environment and Heritage Animal Ethics Committee (number 000214/05) and by the Australian National University Animal Ethics Committee for the southern brown bandicoot sampled at the Timbillica state forests (A2015/26). Samples were collected under a scientific license provided by the NSW Office of Environment and Heritage (number SL100104).

### Sample collection.

Engorged ticks were collected live and immediately frozen in liquid nitrogen during population studies of bandicoots in Sydney’s North Head National Park (33.8224°S, 151.2994°E) and Timbillica state forests (37.3712°S, 149.7211°E) south of Eden, NSW (Fig. 1). Specifically, ticks were collected from two marsupial species: long-nosed bandicoots (Perameles nasuta) in the north (Sydney) and southern brown bandicoots (Isoodon obesulus) in the south (Timbillica). As well as bandicoots, ticks were collected from the eastern blue-tongued lizard (Tiliqua scincoides scincoides) and both native Australian bush rats (Rattus fuscipes) and invasive black rats (Rattus rattus) rats unintentionally caught in treadle cage traps. Unfed I. holocyclus nymphs were collected at Katandra Bushland Sanctuary (33.6744°S, 151.2799°E; Ingleside, Sydney) by flagging during five collection trips between May and September 2017. All the collected ticks were taken to the laboratory and identified using tick taxonomic keys (38).

### Extraction, pooling, and sequencing of RNA.

Ticks were washed in 1× Dulbecco’s phosphate-buffered saline (DPBS) solution and homogenized in 800 μl lysis buffer using a TissueRuptor homogenizer (Qiagen). Total RNA was then extracted using an RNeasy Plus minikit (Qiagen) according to the manufacturer’s instructions. RNA quality was assessed using an Agilent 2100 bioanalyzer (Agilent Technologies). Samples were pooled for sequencing based on criteria including tick species, tick stage, sampling location, and, where possible, the tick host species (Table 1). Host rRNA was first depleted using a Ribo-Zero-Gold rRNA removal kit (human/mouse/rat; Illumina), and sequencing libraries were then prepared using a TruSeq total RNA library preparation kit (Illumina). Paired-end sequencing was performed on the Hiseq2500 platform (Illumina). Library preparation and sequencing were performed by the Australian Genomic Research Facility (AGRF).

### Assembly and analysis.

Sequencing reads were trimmed for quality using a Trimmomatic trimmer and *de novo* assembled using Trinity (v.2.1.1) software (39). The assembled contigs were subjected to BLAST searches against the NCBI nr protein database using the Diamond (v.0.9.10) program (40), and contigs with significant sequence similarity to viral proteins were selected. Significant sequence similarity was determined by an E value of less than 1e−20, a contig length of more than 500 nucleotides, and an amino acid sequence alignment length of more than 100 characters. Sequences were searched for predicted ORFs using the ExPASy Translate tool (https://web.expasy.org/translate/). The predicted ORF structure of the contig was then compared to that of the genome of the closest BLAST hit to determine if the contig was potentially an endogenous virus element rather than a true exogenous virus.

### Identification of nonviral sequences.

The assembled contigs were subjected to BLASTN and BLASTX (v.2.6.0) (41) searches against the NCBI nt and nr databases, and the output was used to identify the key marker gene cytochrome *c* oxidase subunit I (COX1) to confirm the species identification of the ticks in each library. To assess the bacterial, fungal, and single-celled eukaryotic composition of each library, including the presence of B. burgdorferi *sensu lato* bacteria, all contigs showing sequence similarity to the sequences of mammalian or arthropod proteins were excluded from the Diamond BLAST results. The remaining hits were then filtered to include only those contigs showing more than 90% protein sequence similarity over a length of more than 200 amino acids with an E value of less than 1e−20. All results showing homology to ribosomal protein sequences, elongation factor 1 alpha, elongation factor Tu, or cytochrome *c* oxidase subunit 1 were then extracted, and the top BLAST hit was recorded.

### Estimating transcript abundance.

Ribosomal reads were identified by selecting all contigs showing nucleotide sequence similarity of over 90% across a length of over 200 nucleotides and with an E value of less than 1e−20 to ribosomal reads within the NCBI nt database using a BLASTN (v.2.6.0) (41) search. The Bowtie2 (v.2.2.5) program (42) was then used to map sequence reads back to these contigs using the end-to-end alignment algorithm. The Bowtie2 end-to-end alignment was used to map the sequence reads from each library to the viral sequences identified, as well as to the COX1 reference sequence from the host genome. The mapped alignments were then checked in Geneious (v.9.1.5) software (43) to ensure the quality of the alignment and to find any potential mapping errors. The ribosomal read count was then subtracted from the total number of reads when calculating the abundance of viral and COX1 sequences.

### Phylogenetic analysis.

To determine the evolutionary history of each virus, the RNA-dependent RNA polymerase (RdRp) or complete polyprotein of each virus was compared to previously described proteins from the relevant virus family. Accordingly, RdRp or polyprotein sequences representing the family of each virus discovered here were retrieved from the NCBI RefSeq database. The Mafft (v.7.3.0.0) program (44) was then used to align each group of sequences using the L-INS-i algorithm, with all ambiguously aligned regions being removed using the TrimAL (v.1.4.1) program (45). This process resulted in a total of 10 sequence alignments of more than 380 amino acid positions each on the basis of which phylogenetic analysis could proceed. The IQ-Tree (v.1.6.1) algorithm (46) was used to determine the best-fit model of amino acid substitution based on each data set (which was found to be the Le-Gascuel (LG) model in all cases), and maximum likelihood phylogenetic trees were then estimated with the PhyML (v.20150415) program (47), using 100 bootstrapping replications to assess the support for each node.

### Accession number(s).

All consensus virus sequences produced as part of this study have been submitted to GenBank and assigned accession numbers MK026564 to MK026605. All sequence reads are available in the BioProject database under BioProject accession number PRJNA494273.
